# Corrosion mitigation of mild steel in hydrochloric acid solution using grape seed extract

**DOI:** 10.1038/s41598-021-97944-7

**Published:** 2021-09-15

**Authors:** Fatemeh Marhamati, Mohammad Mahdavian, Saeed Bazgir

**Affiliations:** 1 Department of Polymer Engineering Science and Research Branch, Islamic Azad University, Tehran, Iran; 2 Surface Coating and Corrosion Department, Institute for Color Science and Technology, Tehran, Iran

**Keywords:** Corrosion, Metals and alloys

## Abstract

Plant extracts have gained a lot of attention due to their ecofriendly nature for corrosion inhibition. In this study, we examined the inhibition performance of grape seed extract as an eco-environmental inhibitor for mild steel in hydrochloric acid medium. Electrochemical impedance spectroscopy, potentiodynamic polarization, and electrochemical noise techniques were employed to study mild steel's electrochemical behavior in the hydrochloric acid solutions containing grape seed extract. Results depicted that grape seed extract could successfully inhibit the corrosion of mild steel. Besides, water droplet contact angle, field-emission scanning electron microscopy coupled with energy dispersive spectroscopy, Fourier transform infrared spectroscopy, Raman spectroscopy, X-ray photoelectron spectroscopy, and atomic force microscopy were utilized to study the surface of mild steel specimens after dipping in acidic solutions. Electrochemical impedance results showed a corrosion efficiency of about 88% in 300 ppm of grape seed extract. Also, results revealed more compact corrosion products with improved integrity in the presence of grape seed, which confirmed electrochemical test results.

## Introduction

The metallic substrate's corrosion leads to high costs in various industrial sectors. Mild steel (MS) has been extensively utilized as an inexpensive constructional substrate. Due to its low cost and high mechanical performance, it is used in a variety of applications, including chemical and refining processes, petroleum production, construction, and marine applications^1,2^. Various endeavors have been made to reduce the corrosion rate of MS using different methods like coatings, corrosion inhibitors, etc.^3,4^. Corrosion inhibitors have been utilized for diminishing the corrosive influence of acid pickling, cleaning, and descaling of MS in industries.

Venomous organic compounds comprising N, O, and S are extensively used as corrosion inhibitors owing to their prominent protective performance. The protection provided by the traditional organic inhibitors is through an absorption mechanism that prevents contact between the metallic surface and the corrosive environment. However, synthetic organic compounds are non-eco-friendly and expensive, which dramatically reduces their usage for practical applications. Over the last decade, an extensive focus has been given to the so-called “green inhibitors” to overcome the limitations of the traditional synthetic inhibitors due to their cost-effective and environmentally friendly aspects. Green inhibitors have been extracted from the various sections of plants, such as flowers, leaves, stems, roots, shells, seeds, and fruits. They can form a protective film on the metal surface, causing an increase in inhibition efficiency (*IE* %). In other words, they have polar groups containing heteroatoms and aromatic rings that can interact with metal cations (like Fe^2^^+^) on the surface, restricting aggressive ions attacks.

Many plant extracts, including *Lagerstroemia speciosa leaf*^5^, *Persian liquorice*^6^, *Lavandula angustifolia*^7^, *Thymus vulgaris*^8^, *Juglans regia green fruit shell*^9^, *Saraca ashoka*^10^, *Matricaria recutita*^11^, *Tamarindus indiaca*^12^, *Thymus vulgaris*^8^, *Sunflower seed hull*^13^, *Longan seed/peel*^14^, *Allium sativum*^15^, *Salvia officinalis*^16^, *Bambusa arundinacea leaves*^17^, *Eucalyptus leaf*^18^, *Carum carvi*^19^, *Thymus algeriensis*^20^, *Tagetes erecta*^21^, *Esfand seed*^22^*, Turmeric*^23^, *Dacryodis edulis*^24^, *Eriobotrya japonica lindl*^25^, *Egyptian licorice*^26^, *Neem*^27^, *Saffron*^28^, *ginger*^29^, *Valeriana wallichii*^30^, *Menthe pulegium*^31^, have been reported to have corrosion inhibition performance.

**Figure 1:**
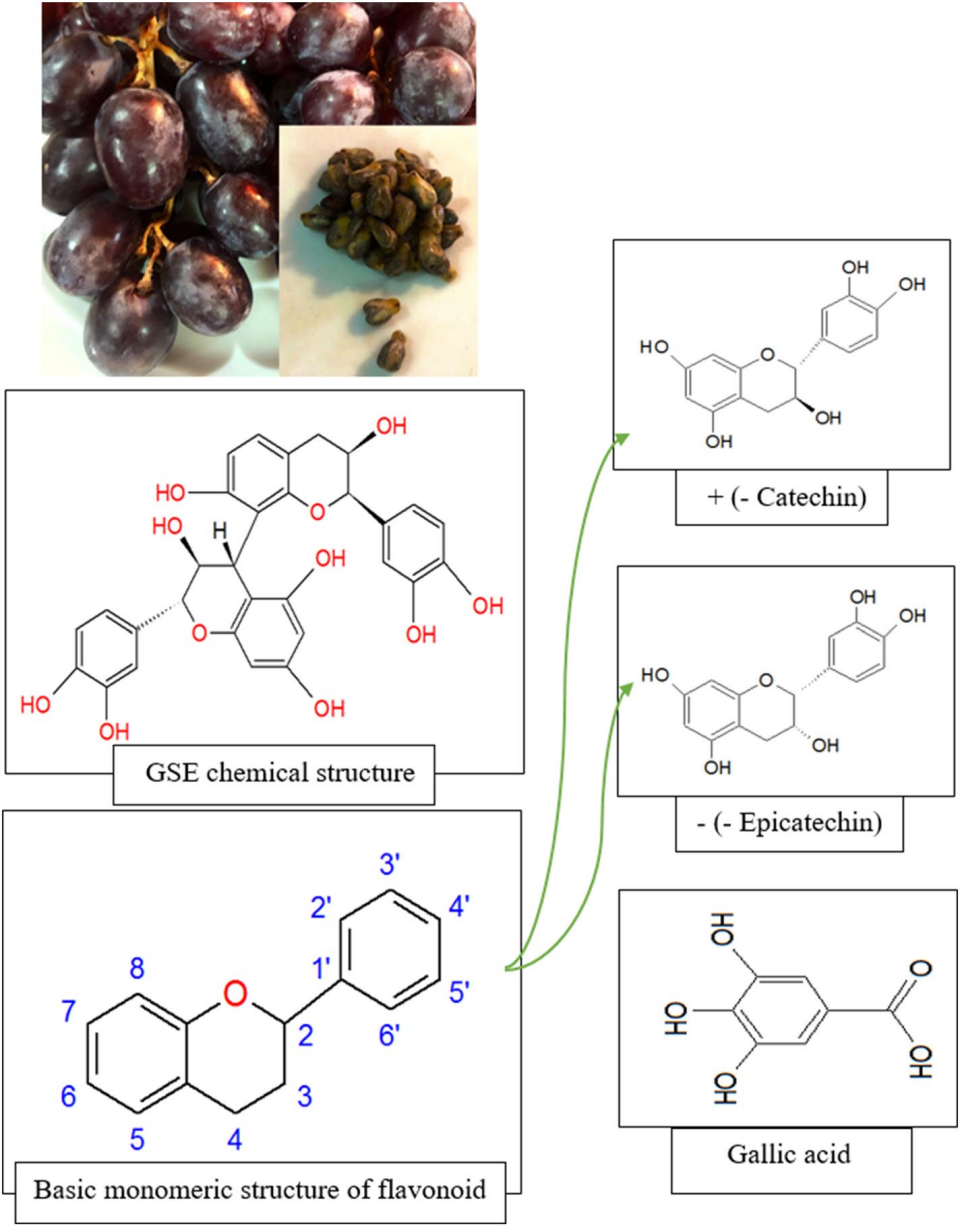
Chemical structure of grape seed extract.

A large amount of grape residue remains in the wine industry every year. *Vitis vinifera* is the prevalent grapevine, domestic to southwestern Asia, from Morocco and Portugal north to southern Germany, the Mediterranean region, central Europe, and east to northern Iran. *Vitis vinifera* is about 5% of the fruit weight, which annually, more than 3 million tons of them are throwing away. Grape seeds have a variety of properties, including the ability to reduce cardiovascular disease, cholesterol, hypertension, and swelling caused by injury, as well as to heal eye diseases and may have anti-cancerous properties. Figure 1 shows some of the main components of the grape seed extract (GSE) containing a significant amount of flavonoids such as catechin, epicatechin, and some phenolic acids.

The corrosion mitigation of MS by GSE has not been divulged so far. Therefore, the corrosion inhibition of GSE as a cheap, eco-friendly by-product of the wine industry was evaluated in this study. Various electrochemical tests have been set up to determine the extent and mechanism of corrosion inhibition.

Moreover, after corrosion experiments, the surface analysis has been applied to prove the interactions of inhibitive species with metal surfaces and/or protective film formation. Furthermore, the specimens' physical chemistry features were evaluated by measuring the contact angle (CA).

## Experimental

### Materials

The GSE was purchased from Ebnemasouye Co. The MS plates (8 cm × 3 cm) were acquired from the Foolad Mobarake Co. The elemental composition of the MS plates is provided in Table 1. Surface preparation was implemented prior to electrochemical tests to eliminate the surface contaminations and attain a convenient surface profile. The MS surface was carefully abraded using sandpapers. Subsequently, the MS plates were sonicated in acetone and methanol to eliminate the surface contaminations. The GSE was added to the hydrochloric acid (1 M) to prepare test solutions (0, 100, 200, and 300 ppm).

**Table 1:** Chemical composition of MS based on weight percentage.

	Fe	Mn	Si	C	Cu	Co	Cr	Mo	P	S
wt%	97.7	1.39	0.415	0.19	0.0481	0.0429	0.026	0.018	0.005	0.005

### Techniques

The potentiodynamic polarization and EIS (electrochemical impedance spectroscopy) were implemented on a 1 cm^2^ exposure area to assess the inhibition efficiency of the GSE. The Ivium software was used to record the electrochemical tests. Zsimpwin software was employed to fit the EIS data. The classical three-electrode system (the working electrode, the Ag/AgCl/3 M KCl reference electrode, and the graphite counter electrode) was used in the electrochemical cell. The EIS measurements were implemented within the frequency range from 10,000 to 0.01 Hz at 10 mV perturbation. The polarization was measured at 1 mV s^−1^ within ± 200 mV (cathodic to anodic side) versus open circuit potential. A cell containing two equal working electrodes and the reference electrode (silver/silver chloride) was utilized to acquire the EN (electrochemical noise) signals. The duration of measurements was 1800s, and the sampling interval was 0.2 s. Triplicate samples were arranged for each electrochemical test to ensure reliability. All the electrochemical measurements were conducted at 25 °C. However, EIS was also carried out at 50 °C.

After exposure to the test solutions, the samples were rinsed with water and dried. The dual scope DME C-26 model of AFM (Atomic Force Microscopy, Semilab Germany GmbH) was used to assess the topography of the exposed samples. The elemental composition and morphology of the surface film formed on the MS specimens subjected to the test solution were assessed by EDS (energy-dispersive X-ray spectroscopy) and SEM analysis. Surface wetting properties were investigated through contact angle measurement using a homemade contact angle measuring system.

Raman confocal and Fourier transform infrared (FTIR) spectroscopies were performed on the MS samples after exposure to the test solutions. After being exposed to the test solution, the film formed on the MS surface was examined by X-ray photoelectron spectroscopy (XPS). XPS (Bes Tec/Germany) was measured in a vacuum chamber of 10^–10^ mbar under 1253.6 eV. Origin 8.0 software was utilized to fit the experimental data with the Gaussian function.

UV–Vis spectroscopy was used to show the emergence of a complex between grape seed extract with iron cations.

## Result and discussion

### Electrochemical performance

#### EIS

**Figure 2:**
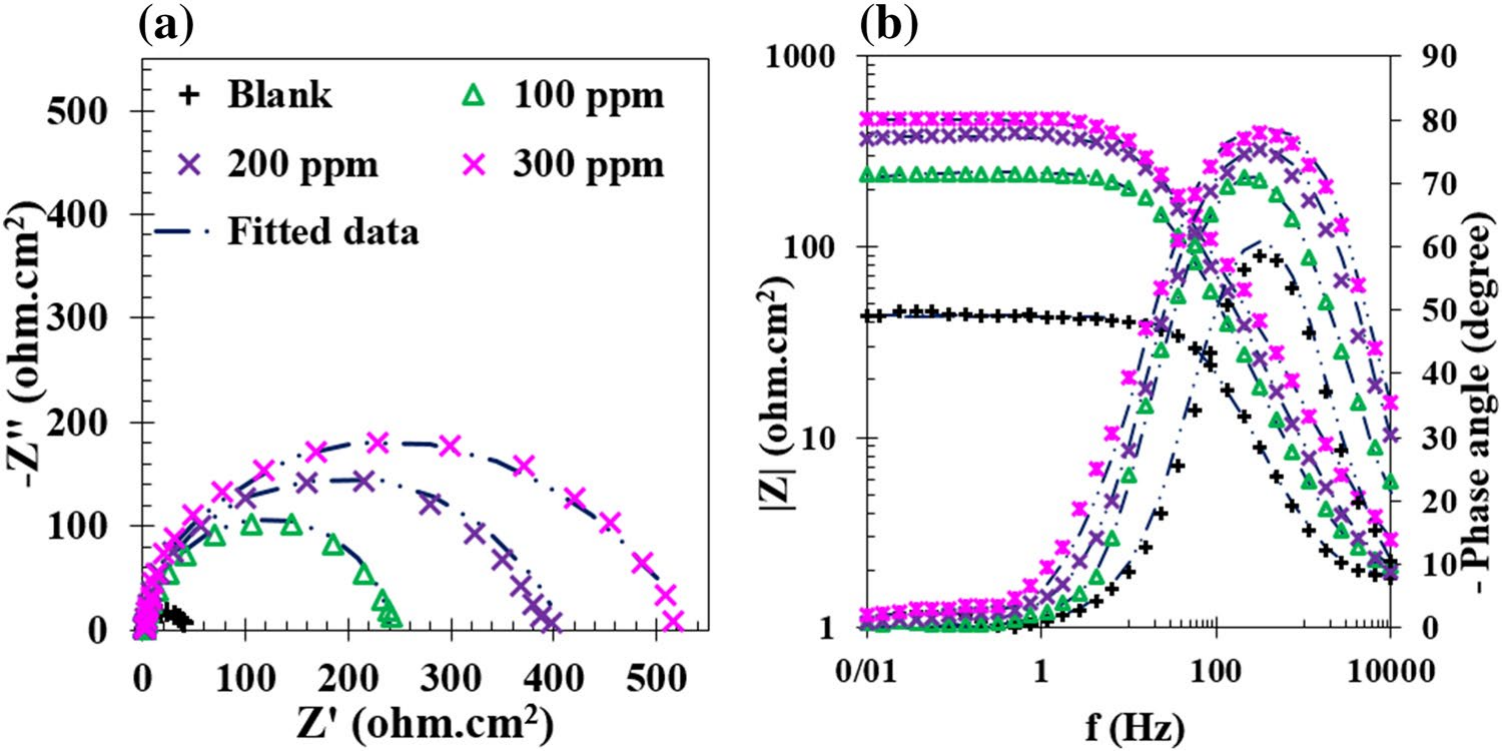
The Nyquist (**a**) and Bode (**b**) diagrams of MS samples immersed in 1 M HCl solution at different concentrations of GSE at 25 °C.

**Figure 3:**
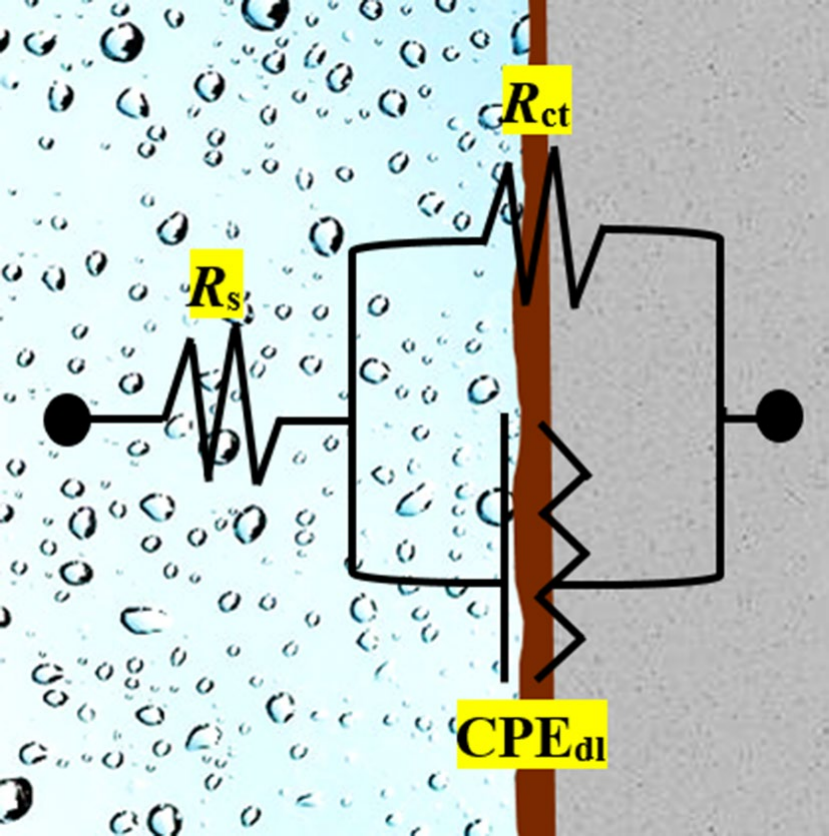
The equivalent electrical circuit employed for fitting EIS data shown in Fig. 2.

EIS was employed to assess the electrochemical properties of MS in the presence and absence of GSE in 1 M HCl solution. Nyquist and Bode diagrams are depicted in Fig. 2. Nyquist curves in Fig. 2a show an increase in the semi-circle diameter with an increment in the GSE concentration. Low-frequency impedance (at 10 mHz) also indicates an increasing behavior with an increase in the GSE concentration, reflecting increasing adsorption of the GSE on the steel surface. The equivalent circuit was employed to fit the EIS data is displayed in Fig. 3. This circuit contains electrolyte resistance (*R*_s_), charge transfer resistance (*R*_ct_), and the constant phase element of the double layer (CPE_dl_). The electrochemical parameters derived from the fittings are provided in Table 2. The effective capacitance of the electrical double layer (*C*_dl_) was calculated using Eq. (1)^32,33^.

1 $${C}_{dl}= {Y}_{0.dl}^{1/n} ({R}_{ct}{R}_{s}/({R}_{ct}+{R}_{s}){)}^{(1-n)/n}$$

where *n* and *Y*_0,dl,_ demonstrate the exponential and the admittance terms of CPE, respectively.

**Table 2:** The electrochemical parameters extracted from EIS data of MS specimens dipped for 3 h in hydrochloric acid solution at different concentrations of GSE at 25 °C.

	CPE			
	R_ct_ (Ω cm^2^)	Y_0_ (Ω^−1^ cm^−2^s^n^)	N	C_dl_ (F cm^−2^)
0 ppm	41.2	1.02 × 10^–4^	0.92	5.84 × 10^–5^
100 ppm	243.9	4.56 × 10^–5^	0.93	2.68 × 10^–5^
200 ppm	392.4	3.56 × 10^–5^	0.92	1.8 × 10^–5^
300 ppm	510.4	5.22 × 10^–5^	0.83	1.23 × 10^–5^

As per Table 2, an increment in the GSE concentration resulted in the decrease of double-layer capacitance and charge transfer resistance increment, indicating an increase in the extent of GSE adsorption on the MS surface.

**Figure 4:**
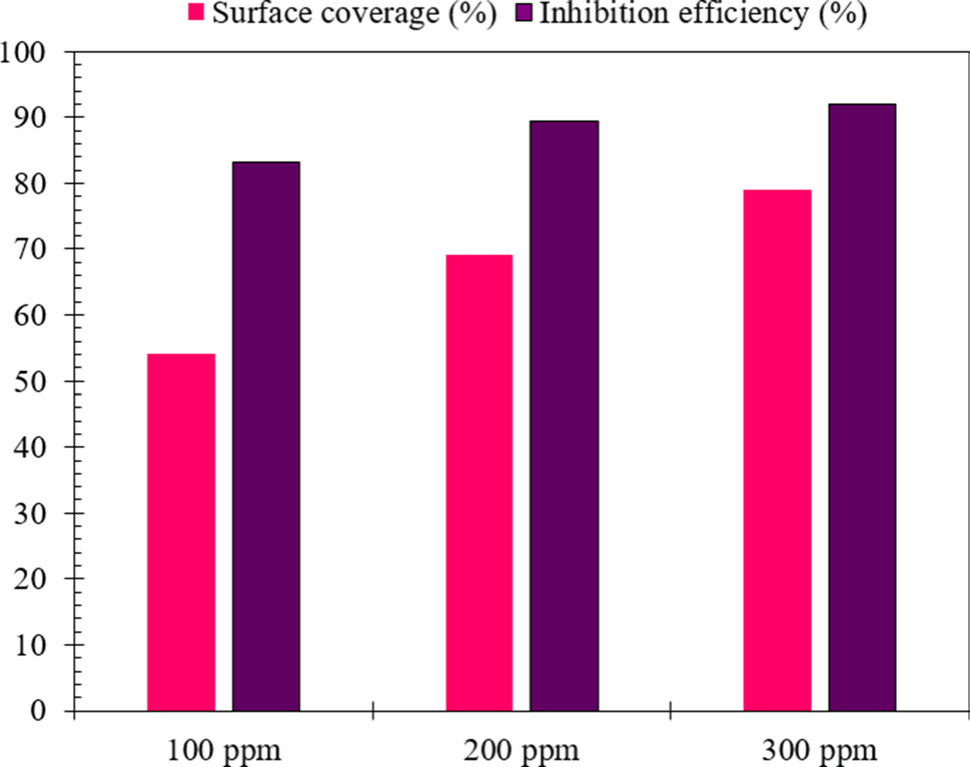
Surface coverage (*θ*%) and inhibition efficiency (*η*%) acquired from EIS data of MS specimens dipped for 3 h in hydrochloric acid solution at different concentrations of GSE at 25 °C.

The inhibition efficiency (*η*) of the grape seed extract was computed using Eq. (2)^34^.

2 $$\eta (\%)={( 1-R}_{ct}/{R}_{ct.i})\times 100$$

where *R*_ct_ and *R*_ct,i_ respectively represent charge transfer resistance in the absence and presence of GSE. Besides, surface coverage (*θ*) was computed based on the variation of double-layer capacitance, according to Eq. (3)^33^.

3 $$\theta \left(\%\right)=100 \times \left(1-\frac{{C}_{dl.i}}{{C}_{dl}}\right)$$

where *C*_dl_ and *C*_dl,i_ respectively represent double layer capacitance in the absence and presence of GSE. The surface coverage and inhibition efficiency results are provided in Fig. 4. The extent of surface coverage reflects the change in the electrical double layer thickness and relative permittivity. In comparison, the extent of inhibition efficiency demonstrates the active surface blocking by the inhibitor. It has been shown that a higher surface coverage than inhibition efficiency indicates the vertical alignment of the inhibitor molecules at the steel-electrolyte interface. In contrast, higher inhibition efficiency than surface coverage discloses horizontal orientation on the inhibitor molecules^33,34^. In this work, higher inhibition efficiency than surface coverage shows effective surface blocking of the GSE molecules by the horizontal alignment on the metal surface.

The effects of temperature changes were investigated on the inhibitory effect of GSE in acidic solution. The results are provided in Supplementary Information (Fig. S1, Fig. S2, and Table S1). Physically adsorbed species usually desorb at elevated temperatures leading to a decrease in inhibition efficiency^35,36^. A glance at these results reveals that by increasing the temperature, the inhibition efficiency of GSE on mild steel was increased, indicating chemisorption of GSE on MS surface.

#### Potentiodynamic polarization

**Figure 5:**
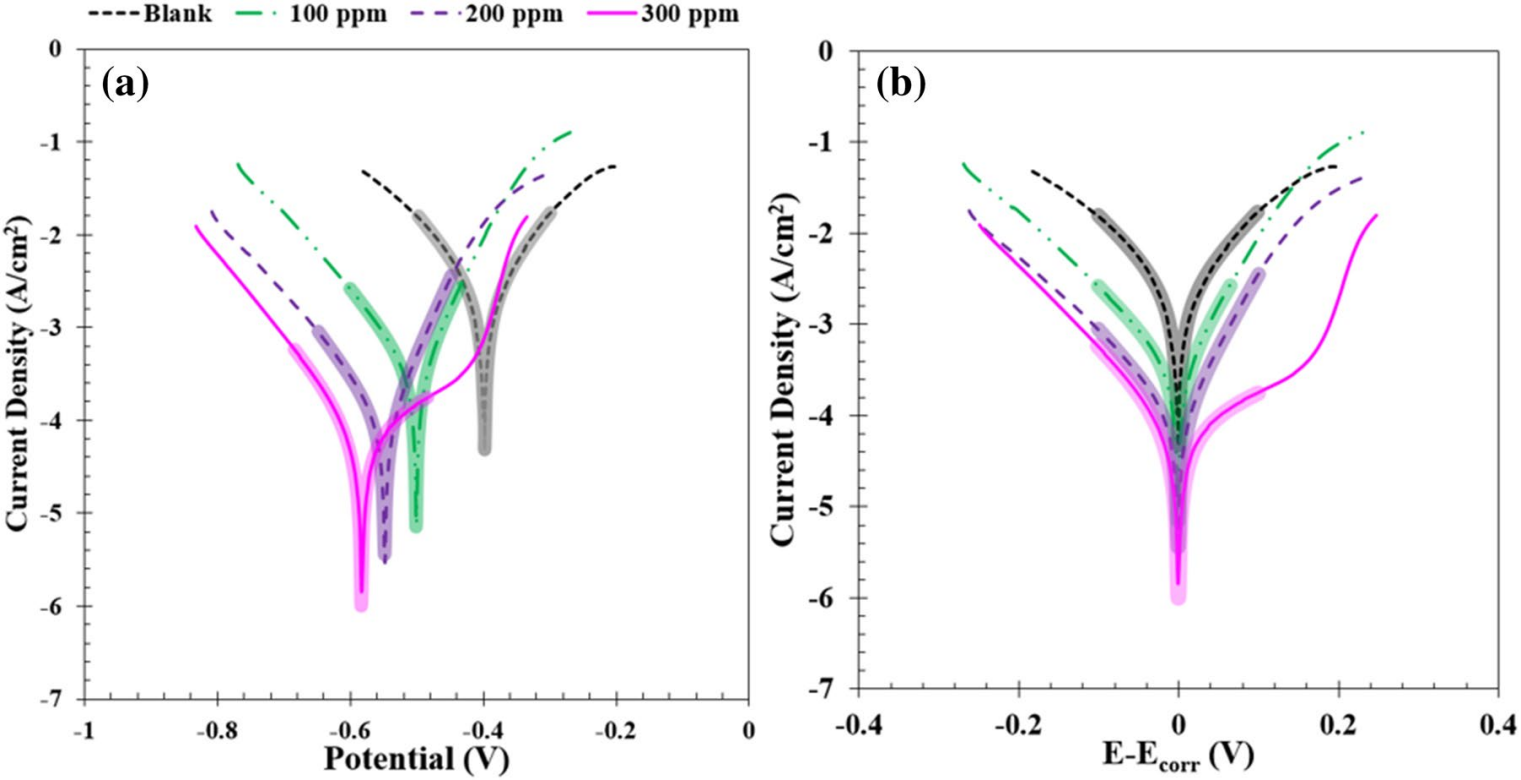
Polarization plot of the MS specimens dipped in HCl solution containing different concentrations of GSE at 25 °C: as recorded (**a**) and shifted to *E*_*corr*_ = 0 V (**b**). Pale-colored curves are fitting curves according to the Wagner–Traud equation.

The corrosion inhibition mechanism of MS dipped for 3 h in hydrochloric acid solution at 0–300 ppm of GSE was assessed by the polarization technique. Figure 5 shows fitted polarization curves of MS immersed for 3 h in the GSE-containing solution. Wagner-Traud equation (Eq. 4) was used to fit the curves and extract the electrochemical parameters^37^.

4 $$i= {i}_{corr}\left[\mathit{exp}\left(\frac{E-{E}_{corr}}{{\beta }_{a}}\right)-\left(\frac{{E}_{corr}-E}{{\beta }_{c}}\right)\right]$$

where the corrosion potential, corrosion current density, cathodic, and anodic Tafel slopes were respectively represented by *E*_corr_, *i*_corr_, *β*_c_*,* and *β*_a_. The inhibition efficiency of the GSE was computed using Eq. (5)^2^.

5 $$\eta = ( 1 - i_{corr} /i_{corr}^{ \circ } )*100$$

where $i_{corr}^{ \circ }$ *i*_corr_ and *i*_corr_ respectively represent corrosion current density in the absence and presence of GSE. All fitted parameters and calculate inhibition efficiency are depicted in Table 3. From this table, it can be discovered that the *i*_corr_ diminished from 2260 μA/cm^2^ for the neat solution (0 ppm GSE) to 87.2 μA/cm^2^ for 300 ppm GSE solution^38^. The *E*_corr_ switches to more negative values by a rise in the GSE concentration. Depression in both cathodic and anodic branches is evident in Fig. 5a. The corrosion potential in the polarization diagrams shifted to zero potential to have a better comparison of the data at the same extent of polarization. The results are given in Fig. 5b. Considering that both anodic and cathodic branches were depressed in the presence of GSE, it can be deduced that this extract mainly acts as a mixed-type inhibitor^32^.

**Table 3:** Electrochemical data obtained from polarization measurements of the MS specimens *dipped* in HCl solution containing different concentrations of GSE at 25 °C.

	E_cor_ (mV)	i_cor_ (μA/cm^2^)	β_a_ (mV/dec)	− β_c_ (mV/dec)	η (%)
0 ppm	− 389	2260	48	51	–
100 ppm	− 500	324	30	47	85
200 ppm	− 547	92.5	27	44	95
300 ppm	− 583	87.2	130	52	96

#### Noise measurement

**Figure 6:**
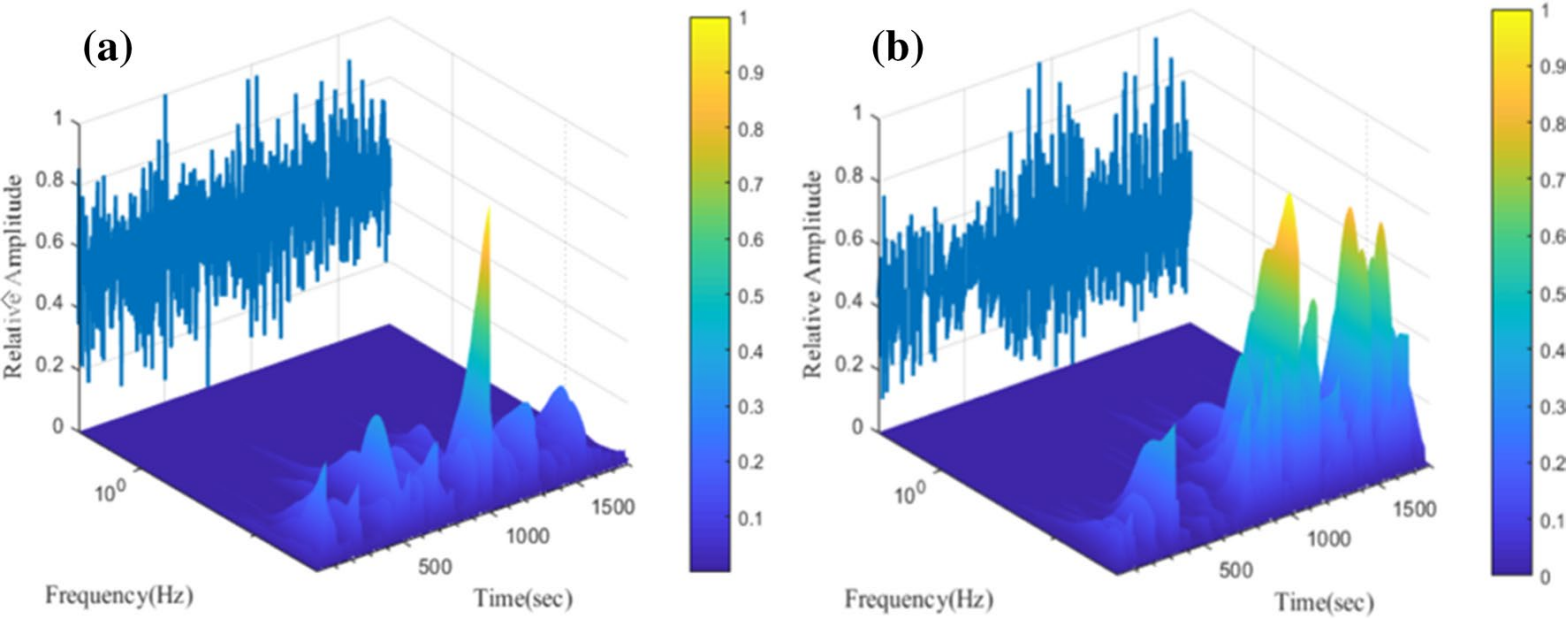
The ECN signals of MS dipped for 3 h in the acid solution containing (**a**) 0 ppm, and (**b**) 300 ppm of GSE.

**Figure 7:**
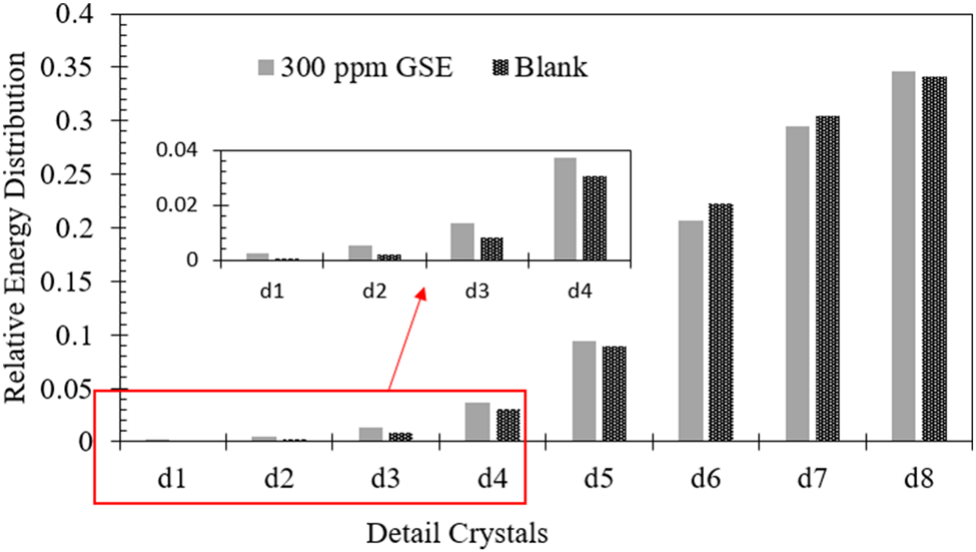
Energy distribution of d-crystals of current noise signals for the MS specimens in the presence and absence of GSE.

The GSE inhibitory effectiveness was also assessed by EN (electrochemical noise) analysis. Figure 6 shows the UWT (undecimated wavelet transform) spectrum of ECN (electrochemical current noise) signals within a span of 1800s, excluding the smooth signals, of the samples dipped for 3 h in 1 M HCl solution in the presence and absence of GSE. The primary ECN signal is shown behind the spectrum with the relative amplitude. From Fig. 6, it is clear that the low-frequency signals in the UWT spectrum of the neat specimen (containing no inhibitor) have higher relative intensities than those in the GSE sample. In Fig. 7, the relative energy distribution of each detail crystal for both specimens is depicted to better assess the contribution of low- and high-frequency transients in the measured ECN. Looking at Fig. 7, the corrosion mechanism for both samples is non-localized general corrosion. However, the relative energy contribution of detail crystals d1–d4 of the GSE sample is more significant than that of the blank specimens meaning that an adsorbed layer of GSE has been formed on the MS surface and the corrosion occurred on the uncovered areas of the adsorbed layer. The total energy of detail crystals (*E*_T_) was measured as per Eq. (6)^6,39^.

6 $${E}_{T}= {\sum }_{j=1}^{8} {\sum }_{k=1}^{n}{d}_{j.k}^{2}$$

The *E*_T_ values were 4.52, 0.21 pA^2^ for blank and GSE samples, respectively. The lower *E*_T_ was acquired for the GSE sample in comparison with the neat one, revealing the lower ECN signal energy in the presence of GSE and, consequently, the inhibitory effect of GSE.

### Surface analysis

#### SEM–EDS

**Figure 8:**
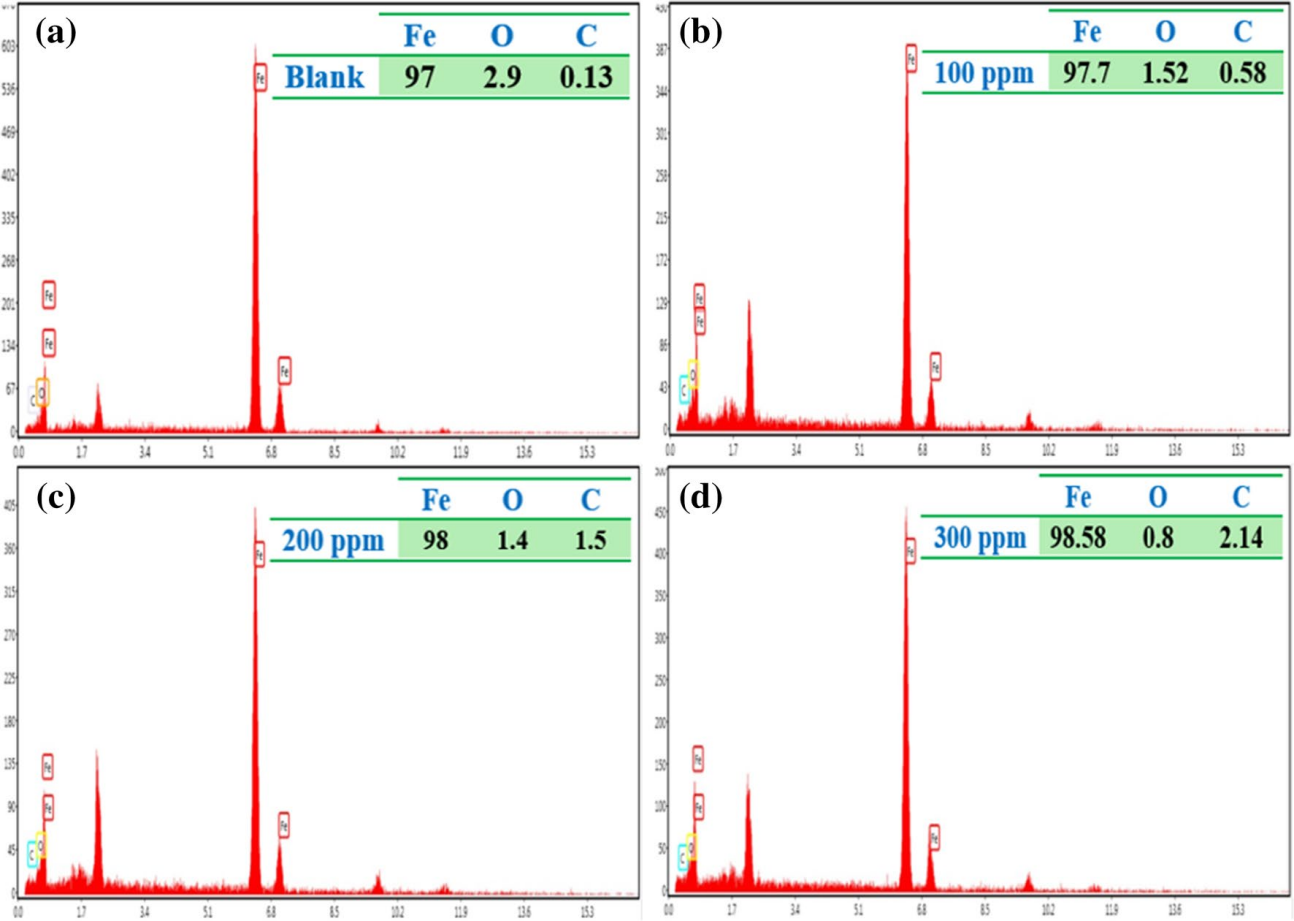
Energy dispersive X-ray (EDX) after exposure to (**a**) 0; (**b**) 100, (**c**) 200, and 300 (**d**) ppm GSE.

**Figure 9:**
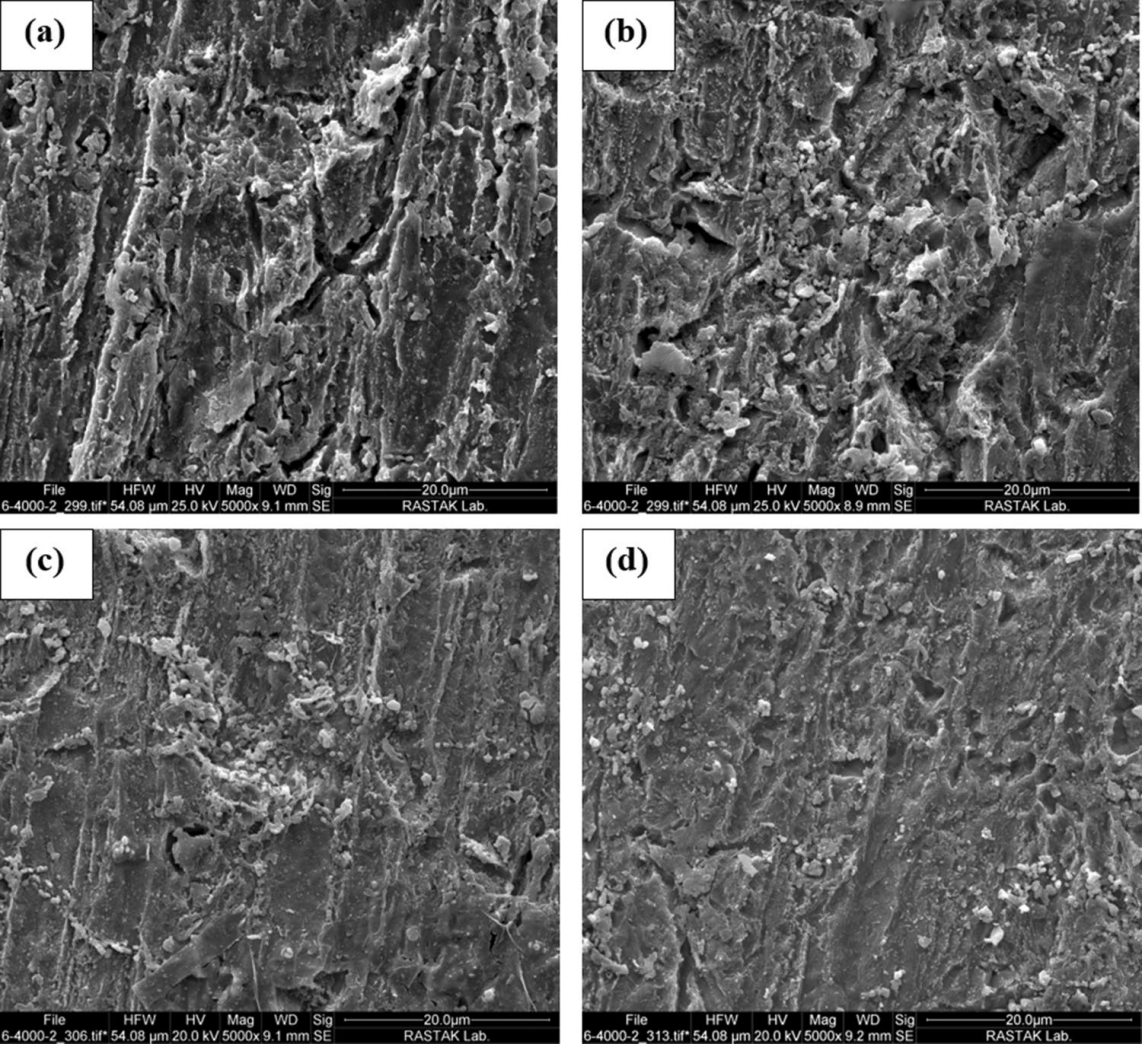
SEM images of MS samples after exposure to (**a**) 0; (**b**) 100, (**c**) 200, and 300 (**d**) ppm GSE.

The specimens' surface film composition subjected to the test conditions for 3 h is provided in Fig. 8. An increment in GSE concentration resulted in a decrement in the percentage of O, revealing a decline in the extent of corrosion. Furthermore, as shown in Fig. 9, an increase in GSE concentration resulted in the establishment of a smoother surface with fewer cracks, indicating less corrosion damage and improved surface film integrity^25,40^. The SEM–EDS results are consistent with the electrochemical test results, in which an increase in the GSE concentration resulted in a reduction in the reaction area, a phenomenon known as the blanketing effect of corrosion inhibitors on the metal surface^41^.

#### AFM

**Figure 10:**
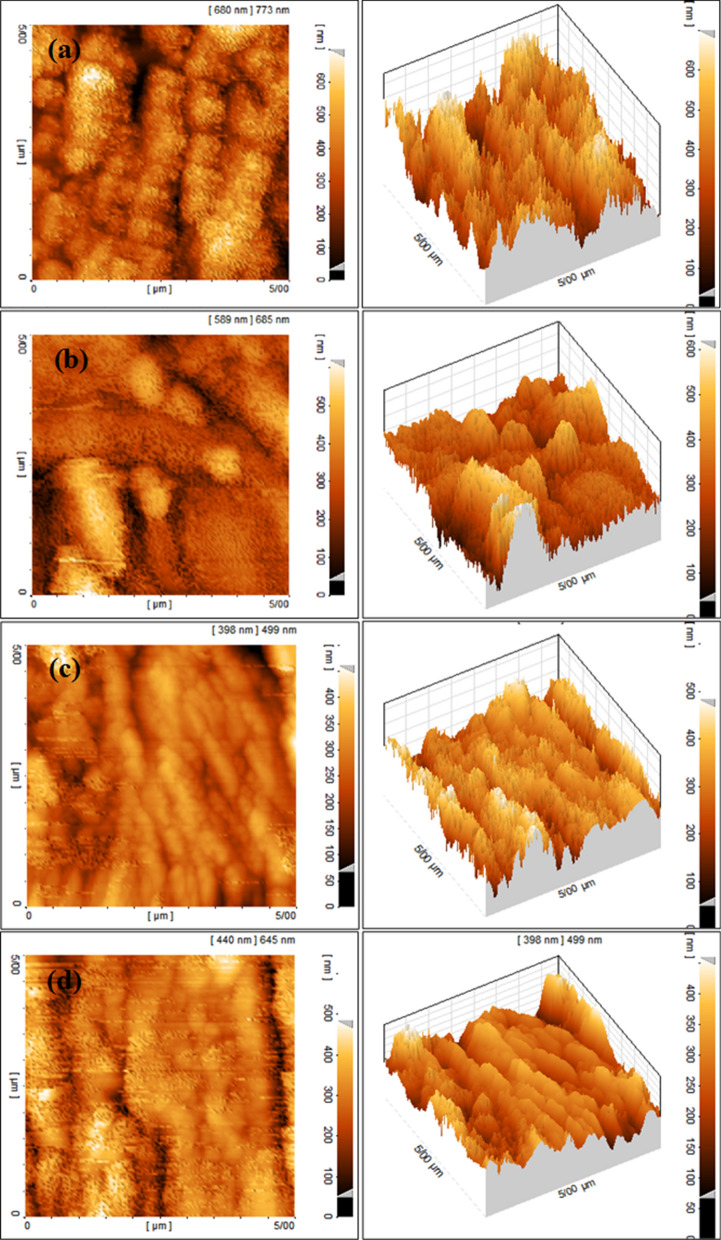
2D and 3D images of AFM from MS immersed for 3 h in the acid solution containing 0 ppm (**a**), 100 ppm (**b**), 200 ppm (**c**), and 300 ppm (**d**) GSE.

According to 3D AFM images provided in Fig. 10, by increasing the concentration of GSE, the surface roughness has been decreased, which is in good accordance with SEM results where a smoother surface was detected at higher concentrations of GSE. A list of the acquired roughness parameters is shown in Table 4. According to this table, the average height distribution (Sa) of the blank sample was 95.3 nm, which was reduced to 72.2, 56.1, and 42.8 nm in the presence of 100, 200, and 300 ppm of GSE, respectively. It has been reported that corrosion inhibitors can reduce the surface roughness^30,31^, which is in good agreement with the outcomes of this work.

**Table 4:** Surface roughness parameters from AFM images from MS after 3 h dipping in the acid solution at different GSE concentrations.

	0 ppm	100 ppm	200 ppm	300 ppm
Sy (nm)	773	685	645	494
Sz (nm)	720	647	522	382
Sa (nm)	95.3	72.2	56.1	42.8

#### Contact angle

**Figure 11:**
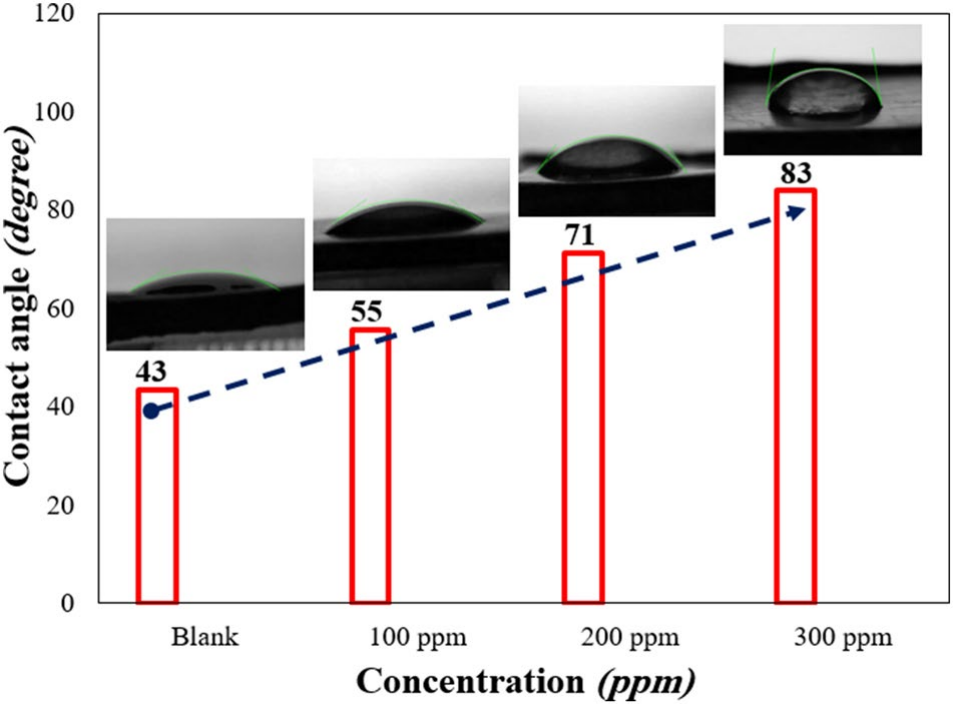
Water droplet contact angle for MS subjected to different concentrations of GSE solutions.

Figure 11 shows the contact angle of water droplets on MS substrate exposed to the test solutions for 3 h. The contact angle of samples showed an increase with an increase in the GSE concentration, reaching the highest at 300 ppm, which was about 83°. The contact angle results indicated that GSE led to an increase in MS surface hydrophobicity, which can be connected to the less oxygen content of the surface layer due to the lower corrosion rate in the presence of GSE^32,37^, which was proved by EDS analysis.

#### FTIR

**Figure 12:**
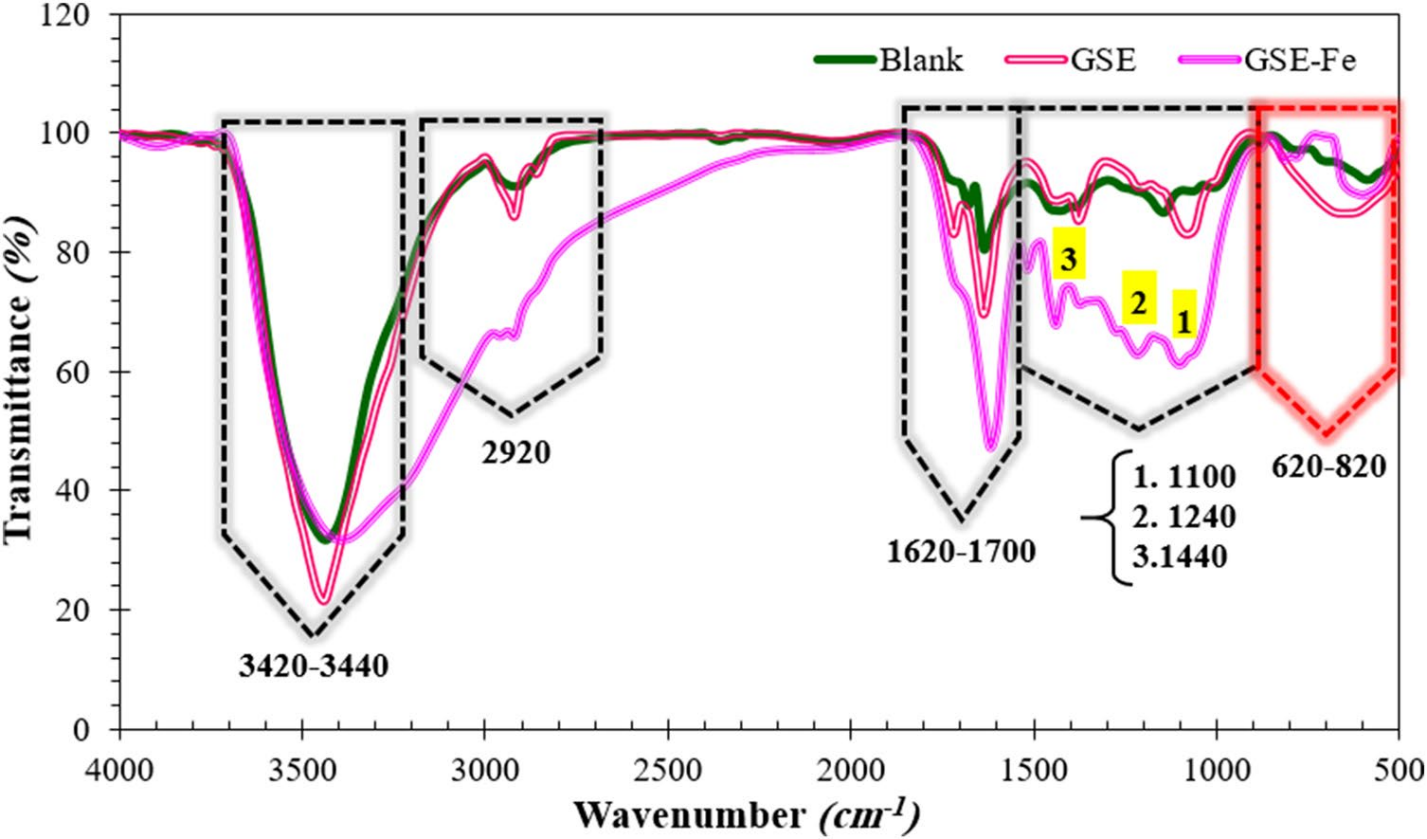
FTIR spectrum of MS immersed for 3 h in the test solution with and without GSE; the GSE-Fe complex is provided as a reference spectrum.

FTIR spectrum test of MS immersed for 3 h in the acid solution in the presence and absence of 300 ppm of GSE are provided in Fig. 12. Also, a complex between Fe^3+^ and GSE was fabricated to assess the chemical composition of mild steel after exposure to the GSE solution. To this end, a mixture of GSE and FeCl_3_ solution at 1:1 weight ratio was prepared in distilled water. The mixture was centrifuged to obtain the settled residue. Finally, the precipitated solid was dried (4 h at 70 °C) and denoted as GSE-Fe. Stretching vibration of hydroxyl groups appeared at 3240–3440 cm^−1^ for all samples^42,43^. The C–H asymmetric stretching vibration occurred at 2920 cm^−1^^42,44^. H_2_O bending vibration for all samples appeared at around 1620 and 1700 cm^−1^, overlapping C=C and C=O stretching vibrations for GSE and GSE-Fe^45,46^. The absorption peaks that occurred at 1100, 1240, and 1440 cm^−1^ are ascribed to C–O stretching vibration, O–H, and C–H bending vibrations, respectively^44,47,48^. Absorption peaks relevant to Fe–O in bending modes appeared below 1000 cm^−1^^45,46^. As seen in Fig. 12, in the presence of GSE as that observed in the GSE-Fe complex, the peak has been shifted to higher wavenumbers confirming the chemical bonding of GSE with the metal surface.

#### Raman

**Figure 13:**
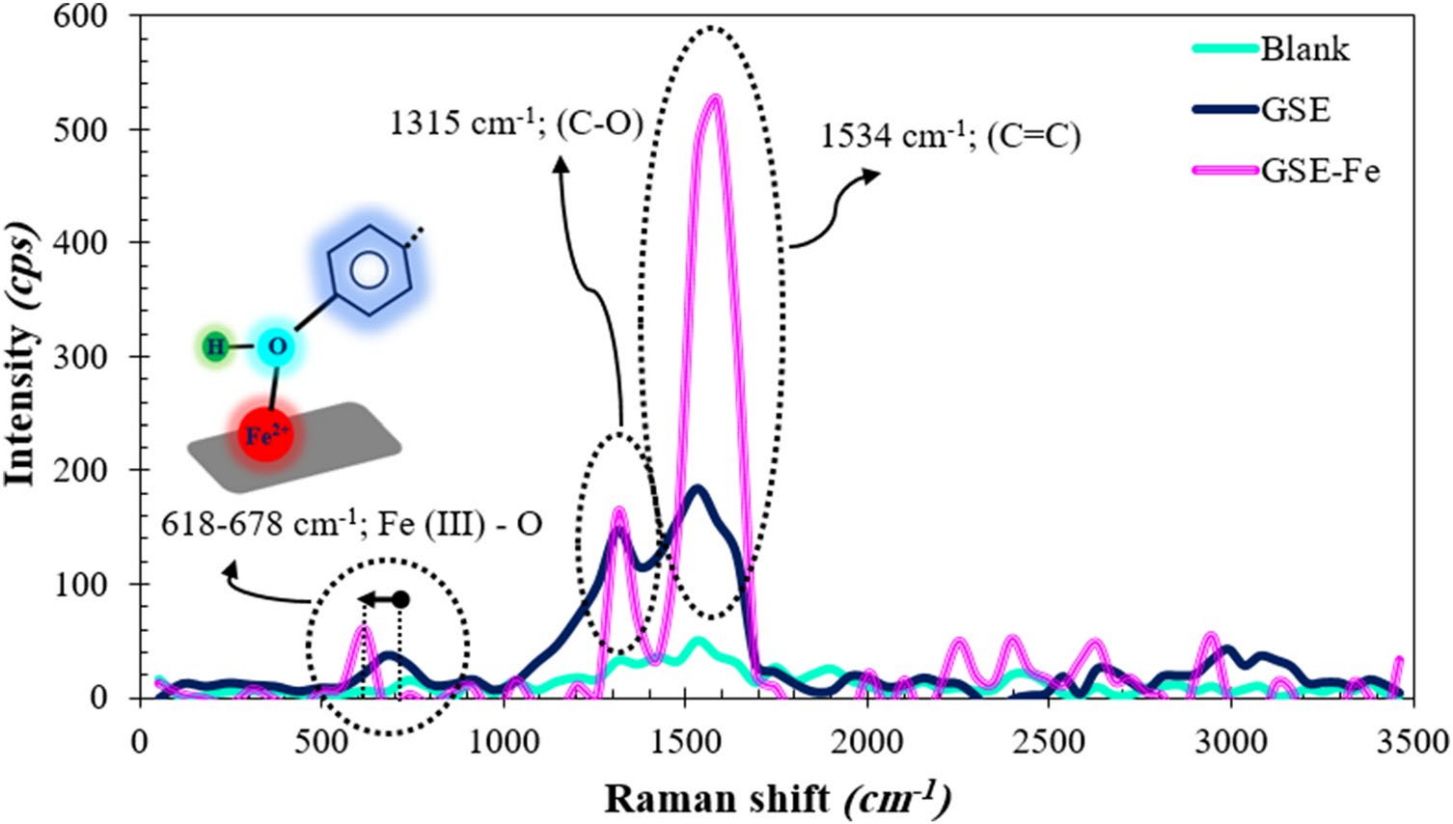
Raman spectroscopy of MS specimens dipped for 3 h in the acid solution with and without 300 ppm of GSE; the GSE-Fe complex is provided as a reference spectrum.

Figure 13 represents the Raman spectra of MS samples exposed for 3 h to the acid solution with and without 300 ppm of GSE. Similar to the FTIR section of the MS, the Raman spectrum after exposure to the GSE solution was compared to that obtained from the GSE-Fe complex. The peaks related to C=O and C=C functions’ stretching vibrations appeared at 1315 and 1534 cm^−1^ attributed to the aromatic rings of GSE structure^48^, which confirmed the adsorption of GSE on the metal surface. The Raman shift observed at 739 cm^−1^ for the blank sample, attributed to Fe–O bond, was shifted to 678 cm^−1^ in the presence of GSE. This peak's shift was evident for GSE-Fe (at 618 cm^−1^), which confirmed the chemical bonding of GSE and MS surface. The Raman results confirmed the FTIR results indicating the chemisorption of GSE on the MS surface.

#### XPS

**Figure 14:**
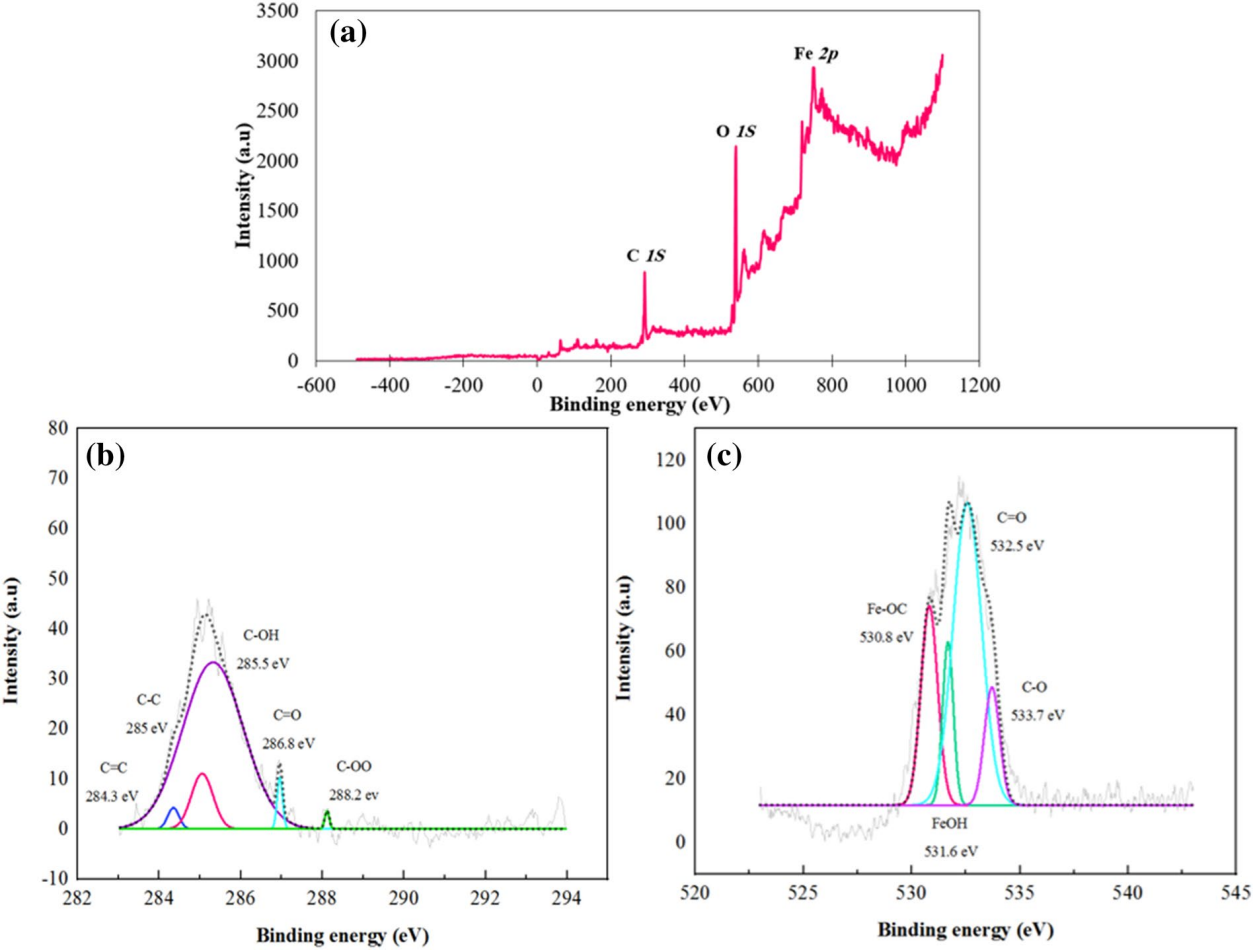
XPS survey spectrum (**a**) along with C *1s* (**b**) and O *1s* (**c**) high-resolution spectra for MS exposed to the GSE solution for 3 h.

The surface analysis of the MS specimens exposed to the GSE solution was also assessed by XPS. Figure 14 depicts the C *1s* and O *1s* high-resolution spectra along with the survey spectrum of mild steel exposed to GSE solution. The survey spectrum (Fig. 14a) indicated the existence of oxygen, carbon, and iron as the main elements. The high resolution C *1s* spectra (Fig. 14b) revealed the peaks at 282.3, 285, 285.5, 286.8, and 288.2 eV attributed to the C=C, C–C, C–OH, C–O–C, and OC=O groups, respectively^49–51^. Figure 14c revealed the peaks at binding energies of 530.8 and 531.6 eV respectively attributed to Fe–OC and Fe–OH bonds on the MS surface, indicating GSE-Fe chemical bonding^52^. In addition, the binding energies at 532.5 and 533.7 eV were respectively connected to C–OH and C–O bonds of the GSE components. The XPS results confirmed the FTIR and Raman results indicating the chemisorption of GSE on the MS surface.

### Interaction of GSE with Fe^3+^ cations

**Figure 15:**
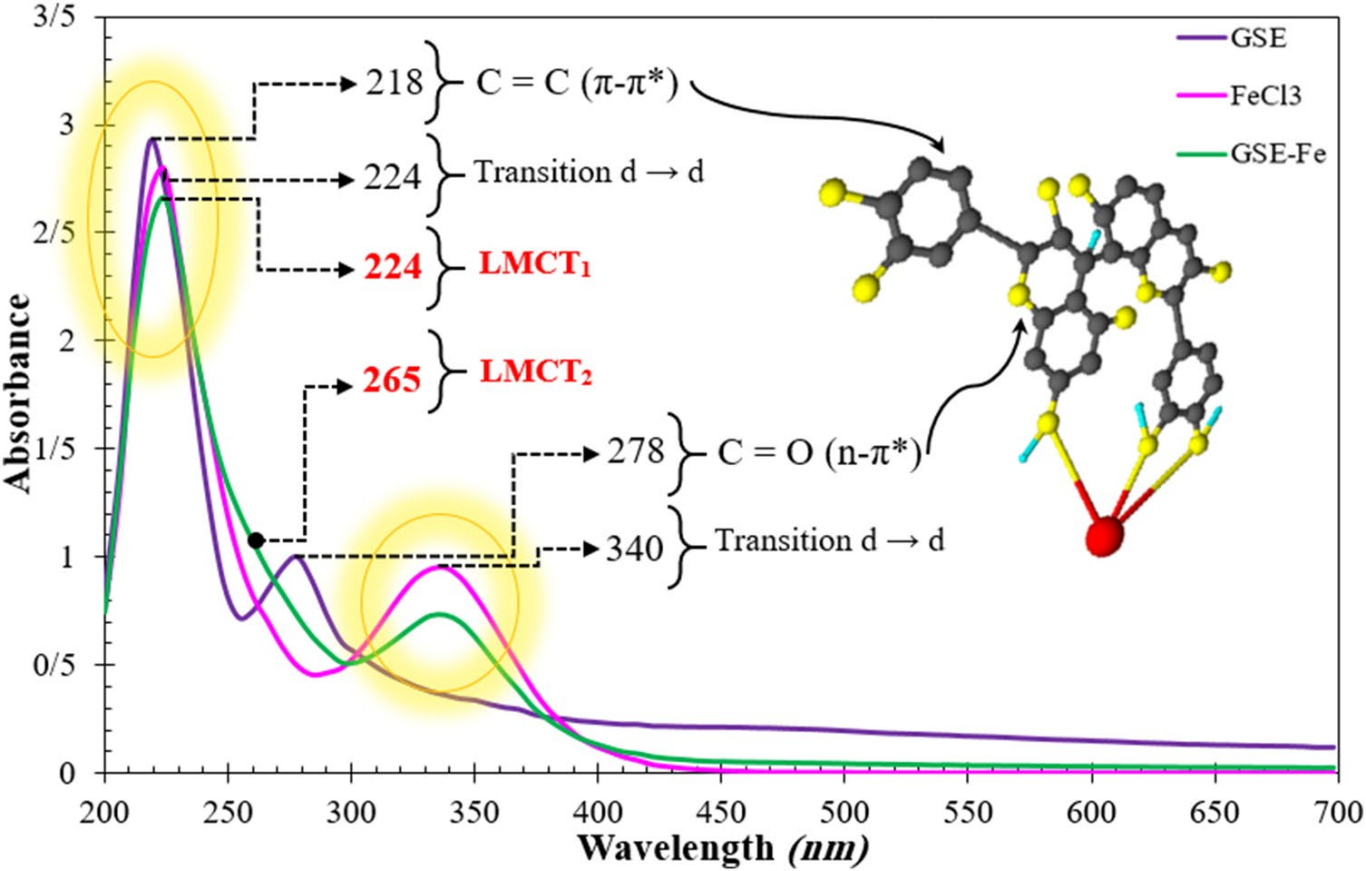
UV–Vis for GSE, metal cations (FeCl_3_), and GSE complex with metal cations (GSE-Fe).

**Figure 16:**
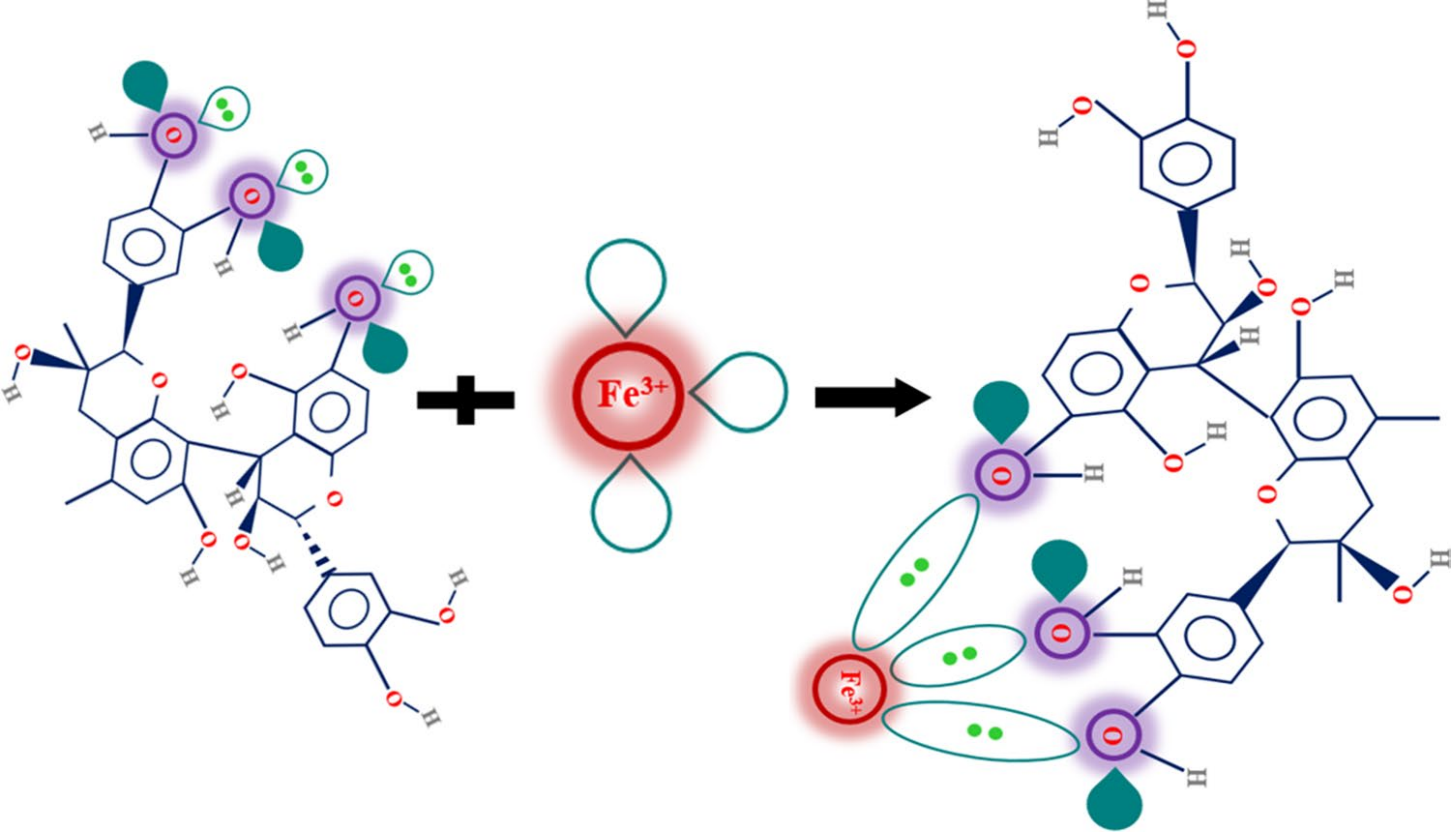
Schematic of complex formation of Fe cation and GSE.

**Figure 17:**
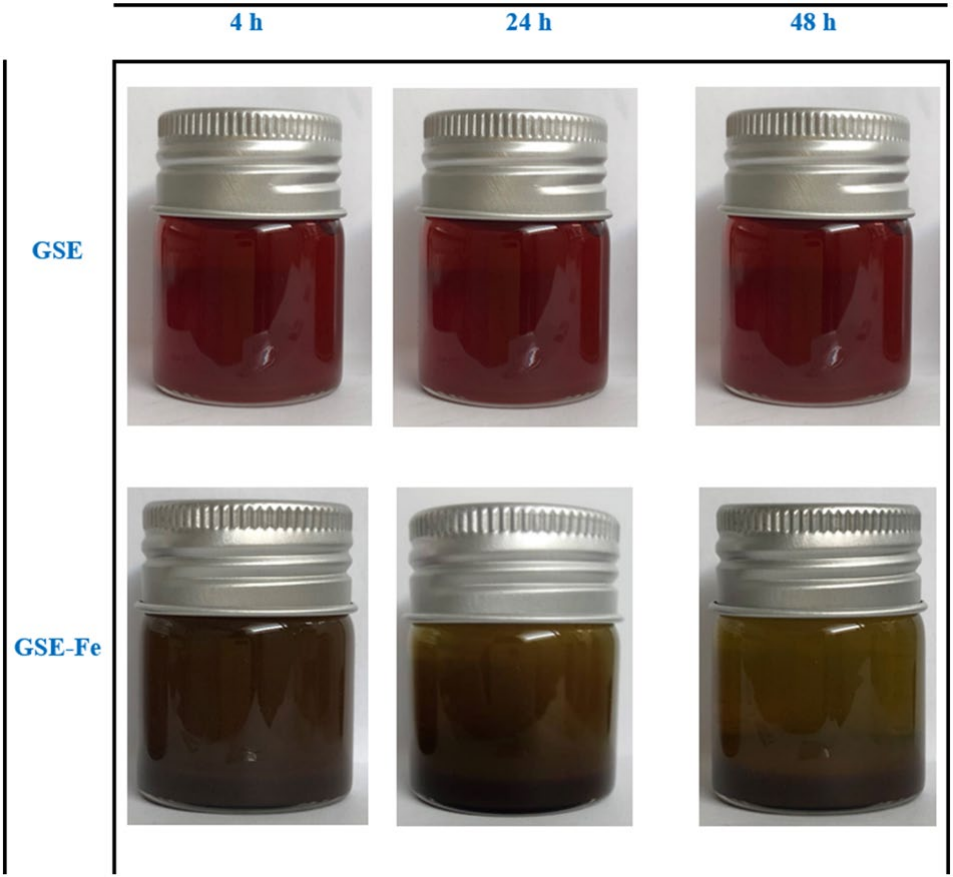
Stability of samples taken from HCl solution and complexes at different interval times.

The possible interaction of GSE with steel surface was scrutinized by UV–Vis analysis in the solution phase between GSE and Fe^3+^ cations. The UV curves are presented in Fig. 15. Looking at this figure, an absorbance peak appeared in 218 and 278 nm regions for GSE solution indicating the existence of π–π* and n–π* transition, respectively, for C=C and C=O organic groups^22,32^. FeCl_3_ solution showed absorption peaks at 224 and 340 nm related to the d-d intra-orbital transitions in ferric cation curves^53,54^. In the presence of Fe3^+^ cations (GSE-Fe), the adsorption bands shifted to 224 nm, indicating the occurrence of LMCT_1_ (the intramolecular transfer of electrons from a ligand to an organic compound which is called Ligand-to-Metal Charge Transfer)^54–57^. Besides, a shoulder formed at 265 nm for the GSE-Fe solution, which broadened the π–π* transition and indicated the complex formation (LMCT_2_). A scheme showing the interaction of GSE and Fe^3+^ cations is provided in Fig. 16. The photo illustrates the coordinated complex between the cation's empty d-orbital with pair of non-bonding electrons on the oxygen atoms in hydroxyl groups. This complex has low solubility, as depicted in Fig. 17. Such complex can be easily formed on the mild steel surface upon exposure to GSE solution. The UV–Vis results showing the chemical bonding of GSE and Fe^3+^ cations confirmed the surface characterization results based on FTIR, Raman, and XPS, indicating the chemisorption of GSE on the MS surface. Furthermore, according to the result of EIS and SEM–EDS, it is clear that the chemisorption of the inhibitor led to the formation of a film of GSE on the metal surface, restricting available reaction areas, know as blanketing effect^41,58,59^.

### Economic study of grape seed extract

Research is ongoing to achieve environmentally friendly and cost-effective inhibitors. Grapes are one of the most popular fruits in the world, and they are primarily used to make wine and fruit juices. Obviously, the resulting waste contains grape skin and seeds. The grape seed was commonly used in the oil industry, but its use increased in the pharmaceutical industries due to antioxidants' presence in its composition. The use of grape seed as a by-product of the alcohol and wine industry makes it very cheap.

In general, grape waste produced in the wine industry is 20%. Due to its high volume as a by-product and valuable substances such as flavonoids, their use as green inhibitors in the corrosion industry is more reasonable than other green inhibitors. Table 5 provides a comparison between green inhibitors in acidic media with grape seed extract. As we studied in this article, grape seed extract's inhibition efficiency at only 300 ppm reaches 92% in an acidic environment, which is exceptional. In fact, most reports have reached a high percentage of inhibition by using a high concentration of inhibitors. Therefore, grape seed extract is a cost-effective corrosion inhibitor for acid inhibition^6^.

**Table 5:** Comparison of the price and inhibition efficiency of the plant extracts in hydrochloric acid solution.

Sort of corrosion inhibitor	Concentration (ppm)	Cost in 1 kg ($)	Max corrosion inhibition (EI %)	Metal	References
Grape seed extract (GSE)	300	30	92	Mild steel	–
Tabernaemontana divaricata	500	229	89.4	Steel	Rose et al.^60^
Morus alba pendula leaves (MAPLE)	4000	129	93	Carbon steel	Jokar et al.^61^
Clove seed	800	35	93	Mild steel	Ali Dehghani et al.^12^
Ginkgo seed	2000	120	90	X70 steel	Qiang et al.^62^
Urtica dioica leaves	800	168	92	Mild steel	Ramezanzadeh et al.^63^
Thymus vulgoris	200	70	62.15	Stainless steel 304	Ehsani et al.^8^
Punica granatum	1000	40	88	Mild steel	Behpour et al.^64^
Glycyrrhiza glabra leaves	800	40	88	Mild steel	Eiman Alibakhshi et al.^40^

## Conclusion

1. GSE was employed as an effective corrosion inhibitor for mild steel in acidic solution. The extract has heteroatoms that can interact with the iron cations on the mild steel surface. FTIR, Raman, and XPS indicated that GSE chemically adsorbs on the MS surface. The formation of a complex between GSE and Fe^3+^ cations was also confirmed by UV–Vis spectroscopy.
2. Increasing the inhibitory concentration and reducing charge transfer confirmed the adsorption of the GSE on the mild steel surface. The maximum inhibition efficiency (η %) in the presence of 300 ppm inhibitor was about 92%. Increasing the temperature revealed an increasing trend in the inhibition efficiency indicating chemisorption of inhibitive species of GSE, which was in accordance with the surface analysis and UV–Vis results.
3. Investigation of surface morphology with and without GSE indicated that corrosion products and surface roughness decreased with increasing inhibitor concentration, indicating improvement in the integrity of the surface film in the presence of GSE.

## Supplementary Information

Supplementary Information.

## References

[1] Wang L (2019). Partially dehydrated zinc hydroxide sulfate nanoplates reinforced coating for corrosion protection. Chem. Eng. J..

[2] Samiee R, Ramezanzade B, Mahdavian M, Alibakhshi E, Bahlakeh G (2019). Graphene oxide nano-sheets loading with praseodymium cations: Adsorption–desorption study, quantum mechanics calculations and dual active-barrier effect for smart coatings fabrication. J. Ind. Eng. Chem..

[3] Fawzy A, Abdallah M, Zaafarany IA, Ahmed SA, Althagafi II (2018). Thermodynamic, kinetic and mechanistic approach to the corrosion inhibition of carbon steel by new synthesized amino acids-based surfactants as green inhibitors in neutral and alkaline aqueous media. J. Mol. Liq..

[4] Zeino A, Abdulazeez I, Khaled M, Jawich MW, Obot IB (2018). Mechanistic study of polyaspartic acid (PASP) as eco-friendly corrosion inhibitor on mild steel in 3% NaCl aerated solution. J. Mol. Liq..

[5] Mobin M, Basik M, El Aoufir Y (2019). Corrosion mitigation of mild steel in acidic medium using Lagerstroemia speciosa leaf extract: A combined experimental and theoretical approach. J. Mol. Liq..

[6] Alibakhshi E (2019). Persian Liquorice extract as a highly efficient sustainable corrosion inhibitor for mild steel in sodium chloride solution. J. Clean. Prod..

[7] Halambek J, Berković K, Vorkapić-Furač J (2010). The influence of *Lavandula angustifolia* L. oil on corrosion of Al–3Mg alloy. Corros. Sci..

[8] Ehsani A (2017). Evaluation of Thymus vulgaris plant extract as an eco-friendly corrosion inhibitor for stainless steel 304 in acidic solution by means of electrochemical impedance spectroscopy, electrochemical noise analysis and density functional theory. J. Colloid Interface Sci..

[9] Haddadi SA, Alibakhshi E, Bahlakeh G, Ramezanzadeh B, Mahdavian M (2019). A detailed atomic level computational and electrochemical exploration of the *Juglans regia* green fruit shell extract as a sustainable and highly efficient green corrosion inhibitor for mild steel in 3.5 wt% NaCl solution. J. Mol. Liq..

[10] Saxena A, Prasad D, Haldhar R, Singh G, Kumar A (2018). Use of *Saraca ashoka* extract as green corrosion inhibitor for mild steel in 0.5 M H_2_SO_4_. J. Mol. Liq..

[11] Nasibi M, Zaarei D, Rashed G, Ghasemi E (2013). Chamomile (*Matricaria recutita*) extract as a corrosion inhibitor for mild steel in hydrochloric acid solution. Chem. Eng. Commun..

[12] Dehghani A, Bahlakeh G, Ramezanzadeh B (2019). Electronic/atomic level fundamental theoretical evaluations combined with electrochemical/surface examinations of *Tamarindus indiaca* aqueous extract as a new green inhibitor for mild steel in acidic solution (HCl 1 M). J. Taiwan Inst. Chem. Eng..

[13] Hassannejad H, Nouri A (2018). Sunflower seed hull extract as a novel green corrosion inhibitor for mild steel in HCl solution. J. Mol. Liq..

[14] Liao LL, Mo S, Luo HQ, Li NB (2017). Longan seed and peel as environmentally friendly corrosion inhibitor for mild steel in acid solution: Experimental and theoretical studies. J. Colloid Interface Sci..

[15] Rodriguez-Clemente E, Gonzalez-Rodriguez JG, Valladares-Cisneros MG (2014). Allium sativum as corrosion inhibitor for carbon steel in sulfuric acid. Int. J. Electrochem. Sci..

[16] Bourazmi H, Tabyaoui M, El Hattabi L, Aoufir YE, Taleb M (2018). Methanolic extract of *Salvia officinalis* plant as a green inhibitor for the corrosion of carbon steel in 1 M HCl. J. Mater. Environ. Sci..

[17] Asipita SA (2014). Green *Bambusa arundinacea* leaves extract as a sustainable corrosion inhibitor in steel reinforced concrete. J. Clean. Prod..

[18] Dehghani A, Bahlakeh G, Ramezanzadeh B (2019). Green Eucalyptus leaf extract: A potent source of bio-active corrosion inhibitors for mild steel. Bioelectrochemistry.

[19] Hattabi LE, Costa J, Desjobert JM, Guenbour A, Tabyaoui M (2016). Electrochemical studies of *Carum carvi* plant as corrosion inhibitor for mild steel in 1M HCl solution. Moroc. J. Chem..

[20] Khadraoui A, Khelifa A, Hachama K, Mehdaoui R (2016). Thymus algeriensis extract as a new eco-friendly corrosion inhibitor for 2024 aluminium alloy in 1 M HCl medium. J. Mol. Liq..

[21] Mourya P, Banerjee S, Singh MM (2014). Corrosion inhibition of mild steel in acidic solution by *Tagetes erecta* (Marigold flower) extract as a green inhibitor. Corros. Sci..

[22] Majd MT, Ramezanzadeh M, Ramezanzadeh B, Bahlakeh G (2020). Production of an environmentally stable anti-corrosion film based on Esfand seed extract molecules-metal cations: Integrated experimental and computer modeling approaches. J. Hazard. Mater..

[23] Edraki M, Mousazadeh Moghadam I, Banimahd Keivani M, Fekri MH (2019). Turmeric extract as a biocompatible inhibitor of mild steel corrosion in 3.5% NaCl solution. Q. J. Iran. Chem. Commun..

[24] Oguzie EE (2010). Adsorption and corrosion-inhibiting effect of *Dacryodis edulis* extract on low-carbon-steel corrosion in acidic media. J. Colloid Interface Sci..

[25] Nikpour S, Ramezanzadeh M, Bahlakeh G, Ramezanzadeh B, Mahdavian M (2019). *Eriobotrya japonica* Lindl leaves extract application for effective corrosion mitigation of mild steel in HCl solution: Experimental and computational studies. Constr. Build. Mater..

[26] Deyab MA (2015). Egyptian licorice extract as a green corrosion inhibitor for copper in hydrochloric acid solution. J. Ind. Eng. Chem..

[27] Parthipan P (2017). Neem extract as a green inhibitor for microbiologically influenced corrosion of carbon steel API 5LX in a hypersaline environments. J. Mol. Liq..

[28] Dob K, Zouaoui E, Zouied D (2018). Corrosion inhibition of curcuma and saffron on A106 Gr B carbon steel in 3% NaCl medium. Anti-Corros. Methods Mater..

[29] Liu Y (2019). Effect of ginger extract as green inhibitor on chloride-induced corrosion of carbon steel in simulated concrete pore solutions. J. Clean. Prod..

[30] Haldhar R, Prasad D, Saxena A, Singh P (2018). Valeriana wallichii root extract as a green & sustainable corrosion inhibitor for mild steel in acidic environments: Experimental and theoretical study. Mater. Chem. Front..

[31] Bouoidina A (2019). Towards understanding the anticorrosive mechanism of novel surfactant based on *Mentha pulegium* oil as eco-friendly bio-source of mild steel in acid medium: A combined DFT and molecular dynamics investigation. Chem. Res. Chin. Univ..

[32] Nematian B, Ahmad Ramazani SA, Mahdavian M, Bahlakeh G, Haddadi SA (2020). Adsorption of eco-friendly carthamus tinctorius on steel surface in saline solution: A combination of electrochemical and theoretical studies. Colloids Surf. A Physicochem. Eng. Asp..

[33] Hirschorn B (2010). Determination of effective capacitance and film thickness from constant-phase-element parameters. Electrochim. Acta.

[34] Mahdavian M (2018). Corrosion of mild steel in hydrochloric acid solution in the presence of two cationic gemini surfactants with and without hydroxyl substituted spacers. Corros. Sci..

[35] Tabatabaei majd M, Bahlakeh G, Dehghani A, Ramezanzadeh B, Ramezanzadeh M (2019). Combined molecular simulation, DFT computation and electrochemical studies of the mild steel corrosion protection against NaCl solution using aqueous Eucalyptus leaves extract molecules linked with zinc ions. J. Mol. Liq..

[36] Mostafatabar AH, Bahlakeh G, Ramezanzadeh B, Dehghani A, Ramezanzadeh M (2021). A comprehensive electronic-scale DFT modeling, atomic-level MC/MD simulation, and electrochemical/surface exploration of active nature-inspired phytochemicals based on *Heracleum persicum* seeds phytoextract for effective retardation of the acidic-induced corrosion of mild steel. J. Mol. Liq..

[37] Frankel, G. S. & Landolt, D. Kinetics of electrolytic corrosion reactions. Encyclopedia of Electrochemistry: Online (2007).

[38] Prabhu D, Rao P (2013). *Coriandrum sativum* L.—A novel green inhibitor for the corrosion inhibition of aluminium in 1.0 M phosphoric acid solution. J. Environ. Chem. Eng..

[39] Mohammadi I, Shahrabi T, Mahdavian M, Izadi M (2020). Sodium diethyldithiocarbamate as a novel corrosion inhibitor to mitigate corrosion of 2024-T3 aluminum alloy in 3.5 wt% NaCl solution. J. Mol. Liq..

[40] Mohammadi M, Shahidi-Zandi M, Foroughi MM (2021). Influence of the dissolved oxygen concentration on the passive oxide film of Al Alloy in different media. Prog. Color Colorants Coat..

[41] Wang M, Zhang J, Wang Q, Du M (2019). Synthesis, characterization and corrosion inhibition performance of the thiourea-chitosan in acidic medium. Int. J. Electrochem. Sci..

[42] Haron W (2013). Structural characteristics and dielectric properties of La_1__−__x_Co_x_FeO_3_ and LaFe_1__−__x_Co_x_O_3_ synthesized via metal organic complexes. Energy Procedia.

[43] Maoela MS (2009). Electroanalytical determination of catechin flavonoid in ethyl acetate extracts of medicinal plants. Int. J. Electrochem. Sci..

[44] Rashid MH, Raula M, Mandal TK (2014). Synthesis of magnetic nanostructures: Shape tuning by the addition of a polymer at low temperature. Mater. Chem. Phys..

[45] Lin Y (2017). Stabilization of arsenic in waste slag using FeCl_2_ or FeCl_3_ stabilizer. RSC Adv..

[46] Markov L, Blaskov V, Klissurski D, Nikolov S (1990). The thermal decomposition mechanism of iron(III) hydroxide carbonate to α-Fe_2_O_3_. J. Mater. Sci..

[47] Somchaidee P, Tedsree K (2018). Green synthesis of high dispersion and narrow size distribution of zero-valent iron nanoparticles using guava leaf (*Psidium guajava* L) extract. Adv. Nat. Sci. Nanosci. Nanotechnol..

[48] Kharazmi A (2015). Structural, optical, opto-thermal and thermal properties of ZnS-PVA nanofluids synthesized through a radiolytic approach. Beilstein J. Nanotechnol..

[49] Mahmudzadeh M, Yari H, Ramezanzadeh B, Mahdavian M (2019). Highly potent radical scavenging-anti-oxidant activity of biologically reduced graphene oxide using Nettle extract as a green bio-genic amines-based reductants source instead of hazardous hydrazine hydrate. J. Hazard. Mater..

[50] Ramezanzadeh M, Ramezanzadeh B, Sari MG, Saeb MR (2020). Corrosion resistance of epoxy coating on mild steel through polyamidoamine dendrimer-covalently functionalized graphene oxide nanosheets. J. Ind. Eng. Chem..

[51] Stuckey JW (2018). Impacts of hydrous manganese oxide on the retention and lability of dissolved organic matter. Geochem. Trans..

[52] Hua Y, Wang S, Xiao J, Cui C, Wang C (2017). Preparation and characterization of Fe_3_O_4_/gallic acid/graphene oxide magnetic nanocomposites as highly efficient Fenton catalysts. RSC Adv..

[53] Zhao R, Pan P (2001). A spectrophotometric study of Fe(II)-chloride complexes in aqueous solutions from 10 to 100°C. Can. J. Chem..

[54] Alorabi AQ, Abdelbaset M, Zabin SA (2020). Colorimetric detection of multiple metal ions using schiff base 1-(2-thiophenylimino)-4-(N-dimethyl)benzene. Chemosensors.

[55] Bahlakeh G, Dehghani A, Ramezanzadeh B, Ramezanzadeh M (2019). Highly effective mild steel corrosion inhibition in 1 M HCl solution by novel green aqueous Mustard seed extract: Experimental, electronic-scale DFT and atomic-scale MC/MD explorations. J. Mol. Liq..

[56] Smit B, Kok J, de Vries L, Dekker F, de Visser B (1999). C hapter^ W. Muscle Nerve.

[57] Bonardi AH, Dumur F, Noirbent G, Lalevée J, Gigmes D (2018). Organometallic vs organic photoredox catalysts for photocuring reactions in the visible region. Beilstein J. Org. Chem..

[58] Soltan A (2021). Effect of corrosion inhibiting compounds on the corrosion behaviour of pure magnesium and the magnesium alloys EV31A, WE43B and ZE41A. J. Magnes. Alloys.

[59] Divakara Shetty S, Shetty N, Parvaz F, Agnihotri K, Mewawala P (2017). Investigating the inhibiting action of thiourea derivative on mild steel corrosion in acid medium. Int. J. Appl. Eng. Res..

[60] Rose K, Kim BS, Rajagopal K, Arumugam S, Devarayan K (2016). Surface protection of steel in acid medium by Tabernaemontana divaricata extract: Physicochemical evidence for adsorption of inhibitor. J. Mol. Liq..

[61] Jokar M, Farahani TS, Ramezanzadeh B (2016). Electrochemical and surface characterizations of morus alba pendula leaves extract (MAPLE) as a green corrosion inhibitor for steel in 1M HCl. J Taiwan Inst Chem Eng.

[62] Qiang Y, Zhang S, Tan B, Chen S (2018). Evaluation of Ginkgo leaf extract as an eco-friendly corrosion inhibitor of X70 steel in HCl solution. Corros. Sci..

[63] Ramezanzadeh M, Bahlakeh G, Sanaei Z, Ramezanzadeh B (2018). Studying the Urtica dioica leaves extract inhibition effect on the mild steel corrosion in 1 M HCl solution: Complementary experimental, ab initio quantum mechanics, Monte Carlo and molecular dynamics studies. J. Mol. Liq..

[64] Behpour M, Ghoreishi SM, Khayatkashani M, Soltani N (2012). Green approach to corrosion inhibition of mild steel in two acidic solutions by the extract of Punica granatum peel and main constituents. Mater. Chem. Phys..

