# Supplementary material for: Corrosion mitigation of mild steel in hydrochloric acid solution using grape seed extract
Source: Sci Rep. 2021 Sep 15;11:18374. doi: 10.1038/s41598-021-97944-7 (PMC8443659; doi:10.1038/s41598-021-97944-7)
Supplement: Supplementary file 1 — Supplementary Information. [file 41598_2021_97944_MOESM1_ESM.docx]

Supplementary information for

Corrosion mitigation of mild steel in hydrochloric acid solution using grape seed extract

Fatemeh Marhamati^a^, Mohammad Mahdavian^b,*^, Saeed Bazgir^a^

*^a^Department of Polymer Engineering Science and Research Branch, Islamic Azad University, Tehran, Iran.*

*^b^ Surface Coating and Corrosion Department, Institute for Color Science and Technology, Tehran, Iran.*

*** Corresponding author. Email: [mahdavian-m@icrc.ac.ir](mailto:mahdavian-m@icrc.ac.ir)


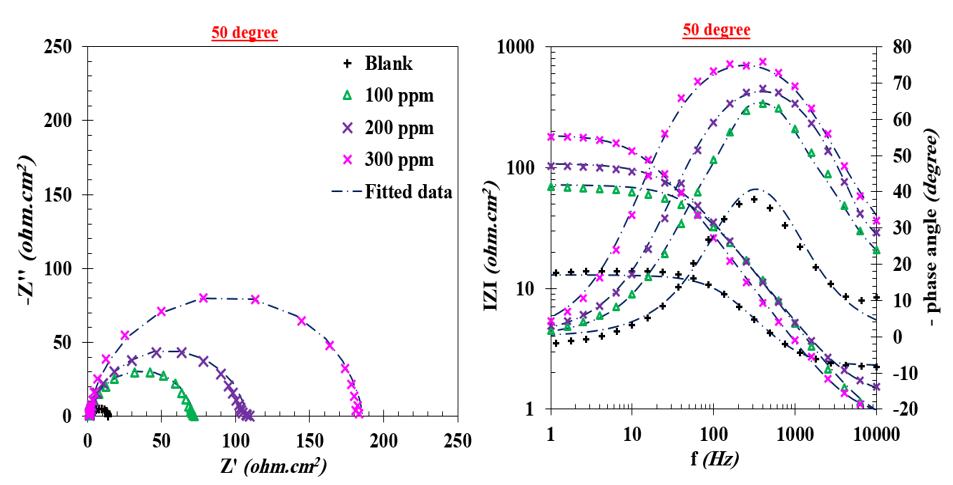
**Fig. S1.** The Nyquist (a) and Bode (b) diagrams of mild steel samples immersed in 1 M HCl solution at different concentrations of GSE at 50°C


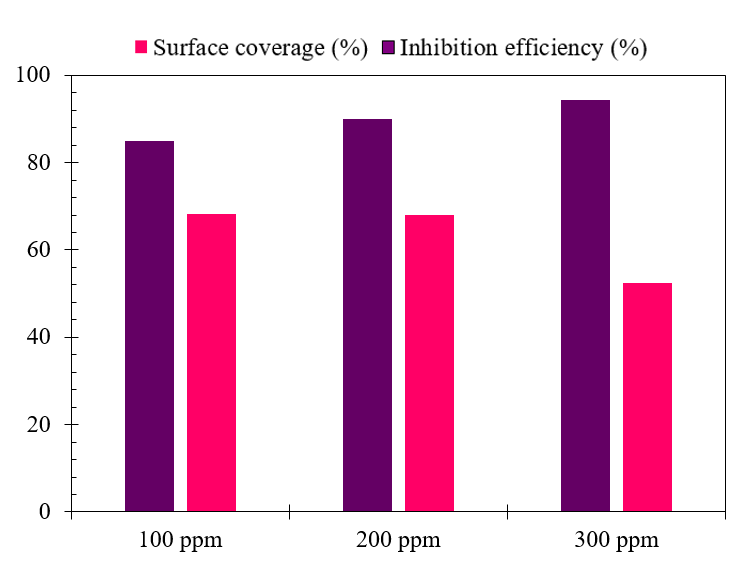


**Fig. S2.** Surface coverage (*θ*%) and inhibition efficiency (*η*%) acquired from EIS data of MS specimens dipped for 3 h in hydrochloric acid solution at different concentration of GSE at 50 °C

**Table S1**. The electrochemical parameters extracted from EIS data of MS specimens dipped for 3 h in hydrochloric acid solution at different concentration of GSE at 50 °C

|  |  | | **CPE** | |  | |  | |
| --- | --- | --- | --- | --- | --- | --- | --- | --- |
| **C_dl_**  **(F cm^-2^)** | **N** | **Y_0_**  **Ω^-1^cm^-2^s^n^))** | | **R_ct_**  **(Ωcm^2^)** | |  | |  |
| 1.08×10^-4^ | 0.92 | | 1.7×10^-4^ | | 10.6 | | **0 ppm** | |
| 3.44×10^-5^ | 0.93 | | 7.07×10^-5^ | | 71.05 | | **100 ppm** | |
| 3.46×10^-5^ | 0.92 | | 0.88×10^-5^ | | 106.5 | | **200 ppm** | |
| 5.14×10^-5^ | 0.83 | | 0.91×10^-5^ | | 185 | | **300 ppm** | |
